# ‘Seeing is believing’: arguing for diagnostic laparoscopy as a diagnostic test for endometriosis

**DOI:** 10.1530/RAF-21-0117

**Published:** 2022-06-10

**Authors:** Jason Mak, Mathew Leonardi, George Condous

**Affiliations:** 1 Acute Gynaecology, Early Pregnancy and Advanced Endosurgery Unit, Nepean Hospital, Sydney Medical School Nepean, University of Sydney, Sydney, New South Wales, Australia; 2 Department of Obstetrics and Gynecology, McMaster University, Hamilton, Ontario, Canada

**Keywords:** diagnostic laparoscopy, endometriosis, surgery, debate

## Abstract

Endometriosis is a benign disease that can cause pain and infertility in women. Debate exists over how endometriosis should best be diagnosed. On one hand, endometriosis can be diagnosed by directly examining pelvic anatomy via a surgical procedure known as diagnostic laparoscopy. On the other hand, the disease can be diagnosed via non-surgical means such as using medical imaging, the symptoms described by the patient and whether the patient responds to non-surgical therapies such as medication. In this debate article, we argue in favour of diagnostic laparoscopy. We review the safety of the procedure, compare the ability of diagnostic laparoscopy vs medical imaging to detect endometriosis and consider the benefits of formally diagnosing or ruling out the condition.

Diagnostic laparoscopy is the process of performing a laparoscopic examination of the pelvis for purely diagnostic purposes, and as indicated in the name, implies that no therapeutic surgical intervention is performed. Diagnostic laparoscopy is a common diagnostic procedure in the workup of patients with pelvic pain, generally attempting to identify endometriosis. A negative diagnostic laparoscopy is done when no endometriosis is identified. A positive diagnostic laparoscopy is done when abnormalities are identified, potentially endometriosis. Additionally, biopsies may be performed as an ancillary histologic diagnostic test (Pascoal *et al.* 2022). In the event endometriosis is identified, patients sometimes go on to have simultaneous surgical treatment. Conversely, there are scenarios where this ‘see and treat’ approach is prevented (Leonardi *et al.* 2018). Factors that might lead to this include whether the surgeon has the ability to perform the operation required or whether the patient is adequately prepared or consented to surgically treat the endometriosis identified. Despite persistent statements that diagnostic laparoscopy is the gold standard method of diagnosing endometriosis (NICE 2017, RANZCOG 2021), advances in non-surgical diagnostic workup and an increasingly risk-averse population have led some to question the validity of diagnostic laparoscopy. Indeed, it has been suggested that diagnostic laparoscopy is obsolete and that diagnostic laparoscopy should be replaced by clinical diagnosis (Agarwal *et al.* 2019). To examine this question, we must evaluate the risks and benefits of the procedure as well as the diagnostic accuracy of non-invasive investigations. We must consider the situations where a diagnostic laparoscopy is justified and whether a negative diagnostic laparoscopy is useful. In this article, we will argue that diagnostic laparoscopy is safe, irreplaceable and a valuable part of the care of women and individuals assigned females at birth with chronic pelvic pain and/or infertility whether pathology is identified or not.

## Argument 1: diagnostic laparoscopy is safe

Overall, laparoscopy is a low-risk procedure. A Cochrane review of entry techniques which included 57 studies and 9865 participants noted that no mortality was recorded in any included studies (Ahmad *et al.* 2019). Around one half of adverse outcomes occur on entry, and this includes vascular injury and visceral injury, both around 3 per 1000 (Ahmad *et al.* 2019). Laparoscopic port site hernia is reported to occur in 0.40–0.66% of procedures, while wound infection has been reported in 0.71% of procedures (Warren *et al.* 2017, Mancini *et al.* 2020). Complications associated with pneumoperitoneum not only include benign and self-limiting shoulder tip pain, which occurs in up to 80% of individuals (Sao *et al.* 2019) and rarer, but also self-limiting surgical emphysema. In the largest cohort study to date of 29,966 laparoscopic surgeries, the overall complication rate was 4.64 per 1000, and mortality was 3.3 per 100,000 (Chapron *et al.* 1998). This cohort demonstrated a direct correlation between surgical complexity and the likelihood of complications. In other words, diagnostic laparoscopy which involves entry and examination only is even safer than the usually quoted risks of laparoscopy. These statistics are general in nature and risk stratification should always be individualized to both the surgeon and patient factors including anticipated surgical complexity.

## Argument 2: identification of endometriosis or other pathology is valuable to patients

While the risks of laparoscopy are low, they must be balanced against the potential benefits. If endometriosis is identified, there is potential for treatment and therefore, improvement in both pain (Sutton *et al.* 1994, Abbott *et al.* 2004, Leonardi *et al.* 2020*a*) and infertility (Roman *et al.* 2018, Moss *et al.* 2021). Furthermore, pathology diagnosed on imaging can be confirmed or additional pathology can be identified. This includes pelvic inflammatory disease, pelvic venous congestion, adnexal pathology, congenital Mullerian anomaly or non-gynecological disease such as appendicitis or diverticular disease.

Diagnostic laparoscopy reduces diagnostic delay and validates patients’ experience of symptoms. In a survey of 451 women, only 10.4% were diagnosed on the first consultation (Lamvu *et al.* 2020). More than half took up to 10 consultations, 7.5% took between 10 and 20 consultations, and 28.4% reported taking more than 20 consultations to reach a diagnosis of endometriosis. A quarter of women reported a diagnostic delay of between 6 and 10 years, while a further quarter reported 11 or more years of delay. Women were most often misdiagnosed as having anxiety, depression or irritable bowel syndrome. Ultimately, 92.5% were confirmed to have endometriosis surgically. These survey data confirm the phenomenon that many gynaecologists have observed: lengthy delays in referral and diagnosis are unfortunately the rule rather than the exception. These delays are also likely to be harmful. Central sensitization is a well-recognized phenomenon in endometriosis, and without diagnosis and intervention, nociceptive dysregulation is exacerbated (Bajaj *et al.* 2003, Stratton & Berkley 2011, Cromeens *et al.* 2021). Similarly, delayed diagnosis of endometriosis has a negative outcome on fertility, given that age is likely to further exacerbate infertility and opportunities for surgical optimization are missed (Cromeens *et al.* 2021). Placing barriers in the way of diagnostic laparoscopy are likely to magnify these challenges.

## Argument 3: a negative diagnostic laparoscopy, ruling out endometriosis, is valuable to patients

Intentionally, we have not included negative diagnostic laparoscopy in our discussion of the risks of laparoscopic surgery. On the contrary, the finding of an absence of endometriosis or other pathology is both inevitable and a valuable outcome in the workup of those presenting with chronic pelvic pain and unexplained infertility. In one published cohort of 255 women undergoing laparoscopy for chronic pelvic pain, 13.7% had a negative result (Jarrell & Arendt-Nielsen 2018). This does not mean that the procedure was in vain. A negative result informs the patient that they do not have endometriosis: an incurable, progressive disease which can cause chronic pain and infertility. In addition, a negative result expedites the additional investigation required to reach a non-endometriosis diagnosis. These alternative diagnoses include pelvic floor dysfunction, allodynia, vaginismus and gut-related pain such as irritable bowel syndrome and interstitial cystitis for example. Referral to gastroenterology, urogynaecology, pain management specialists and pelvic floor physiotherapy can likewise be expedited, and the focus can shift to these new lines of enquiry. It must be stressed that this renewed campaign to accurately diagnose is essential and must be coordinated by the gynaecologist. Failure to do so can lead to delayed diagnosis, feelings of abandonment and the incorrect labelling of the patient as having illness anxiety disorder. In the context of such a failure, the negative diagnostic laparoscopy is ironically reframed as an undesired result because the patient remains without a justifiable explanation for their symptoms.

## Argument 4: diagnostic laparoscopy remains the best method to rule out endometriosis

Ultrasound and MRI can be used to diagnose (i.e. rule in) endometriosis. Biomarkers, patient history and response to medical therapies can increase the suspicion of endometriosis. Whether on their own or in combination, none of these tools has replaced diagnostic laparoscopy, which is still considered the gold standard for diagnosis (NICE 2017, RANZCOG 2021). Several studies have examined the diagnostic accuracy of imaging. A Cochrane review of 49 studies and 4807 participants concluded that MRI and ultrasound were equivalent; however, neither had sufficient diagnostic accuracy to replace surgery for the diagnosis of overall pelvic endometriosis (Nisenblat *et al.* 2016). Imaging has higher diagnostic accuracy for deep than for superficial endometriosis. Using laparoscopy as the gold standard, the sensitivity and specificity for ultrasound detection of deep endometriosis were 79 and 94%, respectively. However, this is an evolving area and new techniques for diagnosing superficial endometriosis are being reported. A recent pilot study with 42 participants demonstrated a significant improvement in diagnostic accuracy for superficial endometriosis, when a specialized technique was employed (Leonardi *et al.* 2020*b*). When excluding those with more advanced forms of endometriosis, the diagnostic performance was as follows: sensitivity 77.7%, specificity 100.0%, positive predictive value 100.0% and negative predictive value 33.3%. In general, high PPV infers that disease identified on imaging is sufficient for diagnostic purposes. This is relevant as any subsequent laparoscopic procedure should be planned and consented accordingly. Conversely, a low NPV infers that the absence of disease on imaging does not rule it out, and diagnostic laparoscopy is still required for diagnosis.

The anatomical location of deep endometriosis is a key variable when considering its diagnostic accuracy. A series of three meta-analyses demonstrate this (Gerges *et al.* 2021*a*,*b*,*c*). Rectosigmoid disease has the highest sensitivity, followed by uterosacral ligament, vaginal, rectovaginal septum and then bladder deep endometriosis. The sensitivities are 86–89, 60–81, 52–64, 57 and 55%. Where a range is quoted, a difference between transvaginal sonography (TVS) and MRI was detected. MRI was superior for uterosacral and vaginal disease, while TVS was superior for rectosigmoid disease. Across all locations and modalities, specificity was excellent, ranging from 95 to 100%. Another similar systematic review specifically examined deep endometriosis. Again, MRI and ultrasound performed equally well; however, accuracy depended on location. Again, rectosigmoid disease had the highest sensitivity at 85% for both modalities (Guerriero *et al.* 2018).

While imaging is improving our diagnostic rate of endometriosis pre-operatively, historically and still in settings where advanced imaging techniques are available, most abnormalities that are discovered at laparoscopy are not identified in pre-operative workup at all. In a cohort of 48 women with chronic pelvic pain, 98% had pathology that was not identified during pre-operative history, examination or imaging (Brichant *et al.* 2018). Another cohort of 120 women was admitted to the hospital under the care of the gynaecology team with an uncertain diagnosis after 4 weeks. Despite the assistance of imaging, more than half of these cases had new diagnoses following a diagnostic laparoscopy (Nar *et al.* 2014). Likewise, a cohort of 100 women who underwent laparoscopy by a gynaecologist for acute abdomen found that 44% had an incorrect pre-operative diagnosis (Cohen *et al.* 2001). Therefore, there is good evidence to support the assertion that diagnostic laparoscopy plays a very important part in the diagnosis, not just of endometriosis but of other pain presentations in gynaecology more broadly.

Just as imaging should not replace laparoscopy, nor should laparoscopy replace imaging. The Ultrasound-Based Endometriosis Staging System (UBESS) has been temporally and externally validated to accurately predict the surgical complexity level encountered at laparoscopy (Menakaya *et al.* 2016, Tompsett *et al.* 2019, Espada *et al.* 2021). Ultrasound, therefore, has a vital role to play in pre-operative triage, in making sure an appropriately skilled surgeon is performing the laparoscopy, and the patient has been adequately consented and prepared for the anticipated pathology. Indeed, it is important that the surgeon performing the laparoscopy has the ability to ‘see *and* treat’ disease with the highest level of surgical complexity and to adequately survey the pelvis and abdomen. Expertise is required as lesions can be subtle, occult or atypical. A cohort of 61 women who had been referred to a specialist centre after a negative diagnostic laparoscopy underwent repeat laparoscopy. A quarter of these women were found to have occult posterior compartment endometriosis that was previously not identified (Griffiths *et al.* 2007). This study may simply highlight the operator-dependent diagnostic nature of diagnostic laparoscopy, which is shared among all diagnostic tests (Pascoal *et al.* 2022).

The diagnostic accuracy of UBESS increases as the severity of the disease increases, with the highest level of accuracy found with deep endometriosis (Nisenblat *et al.* 2016). By happy coincidence, this corresponds to the potential for a diagnostic laparoscopy to miss the deep disease. Goncalves and colleagues have shown that for vaginal and rectosigmoid endometriosis, diagnostic laparoscopy had lower sensitivity and specificity than TVS (Goncalves *et al.* 2021). This highlights the potential for a diagnostic laparoscopy and imaging to complement one another. While further research is needed, laparoscopy may not be the gold standard when it comes to diagnosing endometriosis in some locations. Endometriosis is already notorious for delayed diagnosis;therefore, a false negative diagnostic laparoscopy compounds what is already a harrowing patient journey.

The recent European Society of Human Reproduction and Embryology (ESHRE) 2022 endometriosis guidelines recommend that empirical (pharmacological) treatment can be considered in place of diagnostic laparoscopy (ESHRE 2022*a*). This is significant divergence from antecedent guidelines. It should be noted that there is no clear empirical evidence for this statement, and supporting citations consist of three opinion pieces (ESHRE 2022*b*). The accompanying review report reveals an apparent risk of bias, whereby the main proponents of empirical therapy are the pharmaceutical company representatives who contributed to the document. In addition, of the 15 independent reviewers, 9 list pharmaceutical company funding in their disclosures (ESHRE 2022*c*). Response to empirical therapy should not be considered diagnostic. Just as laparoscopy is not mandatory in all cases of endometriosis, empirical treatment does not replace diagnosis or exclude laparoscopy for diagnosis or treatment. Care should be individualized and the informed choice of the patient should be supported.

## Argument 5: diagnostic laparoscopy is valuable for the infertile patient

Endometriosis is a double-edged disease. Alongside pain, infertility is also an important implication. Whether excision of endometriosis improves fertility outcomes is still highly debated (Gordts 2021, Leonardi 2021) and that debate should not be confused with the value of diagnosis. What is not controversial is the fact that endometriosis has a very strong association with infertility. In women undergoing laparoscopy for unexplained infertility, 60% are found to have endometriosis, making it a high-yield diagnostic tool (Pantou *et al.* 2019). A retrospective cohort study of 1322 women using self-reported outcomes found that one-third of women undergoing assisted reproductive technologies (ART) had a diagnosis of endometriosis. It also identified an interesting difference between women who were diagnosed with endometriosis before vs after commencing ART. Women who were diagnosed after commencing ART required more *in vitro* fertilization cycles and were less likely to report a birth than women who were diagnosed with endometriosis before commencing ART (Moss *et al.* 2021). Whether endometriosis is identified or not, diagnostic laparoscopy provides valuable information for the infertile couple.

## Conclusion

For the sufferer of chronic pelvic pain or the infertile couple, diagnostic laparoscopy provides the answers that are desperately sought. Whether endometriosis is diagnosed or not and whether treatment is triggered or not are irrelevant to this debate. The reality is laparoscopy is safe and is irreplaceable. We have argued that diagnostic laparoscopy plays a critical role in diagnosing endometriosis, but the surgeon should never fly blind. Pre-operative assessment with history-taking, physical examination and imaging provides an important triage and clinical decision-making role.

While the benefits of a positive laparoscopy are obvious, the importance of a negative laparoscopy is often an undervalued key step in redirecting investigations and treatment. Despite advances in diagnostic imaging for endometriosis, the data demonstrate the disease cannot be ruled out until the pelvis and abdomen are directly visualized, with biopsies taken of abnormal areas. Diagnostic laparoscopy is not yet antiquated. While it should not be considered mandatory, it remains the gold standard for diagnosis and an important gateway to treatment.

## Declaration of interest

The authors declare that they are both gynaecology surgeons and sonologists: distinctiona that carry mutually exclusive risks of bias in this debate. J M has no funding or sponsorship to report. M L reports grants from OZWAC, Endometriosis Australia, AbbVie, CanSAGE, MRFF, HHS; honoraria for lectures/writing from GE Healthcare, Bayer, AbbVie, TerSera, consulting fees from Imagendo, outside the submitted work. G C reports grants from Endometriosis Australia and MRFF; honoraria for lectures from G E Healthcare and Imperial College London. Mathew Leonardi is an Associate Editor of Reproduction and Fertility. Mathew Leonardi was not involved in the review or editorial process for this paper, on which he is listed as an author.

## Funding

This work did not receive any specific grant from any funding agency in the public, commercial or not-for-profit sector.

## Author contribution statement

All listed authors contributed to the conceptualization, writing and editing of this piece.
